# Efficacy of metformin and fermentable fiber combination therapy in adolescents with severe obesity and insulin resistance: study protocol for a double-blind randomized controlled trial

**DOI:** 10.1186/s13063-021-05060-8

**Published:** 2021-02-17

**Authors:** Edward C. Deehan, Eloisa Colin-Ramirez, Lucila Triador, Karen L. Madsen, Carla M. Prado, Catherine J. Field, Geoff D. C. Ball, Qiming Tan, Camila Orsso, Irina Dinu, Mohammadreza Pakseresht, Daniela Rubin, Arya M. Sharma, Hein Tun, Jens Walter, Christopher B. Newgard, Michael Freemark, Eytan Wine, Andrea M. Haqq

**Affiliations:** 1 Department of Agricultural, Food and Nutritional Science, University of Alberta, Edmonton, T6G 2E1 AB Canada; 2 Department of Pediatrics, University of Alberta, Edmonton, T6G 2E1 AB Canada; 3 Department of Medicine, University of Alberta, Edmonton, T6G 2C2 AB Canada; 4 School of Public Health, University of Alberta, Edmonton, T6G 1C9 AB Canada; 5 California State University Fullerton, Fullerton, USA; 6 University of Hong Kong School of Public Health, Hong Kong, China; 7 DNational University of Ireland University College Cork, University College Cork, Cork, Ireland; 8 Duke University Medical Center, Duke University Hospital, Durham, NC USA; 9 Department of Pediatrics and Physiology, University of Alberta, Edmonton, T6G 1C9 BA Canada

**Keywords:** Adolescents, Insulin resistance, Diabetes, Obesity, Metformin, Dietary fiber, Gut microbiome

## Abstract

**Background:**

Accumulating evidence suggests that the metabolic effects of metformin and fermentable fibers are mediated, in part, through diverging or overlapping effects on the composition and metabolic functions of the gut microbiome. Pre-clinical animal models have established that the addition of fiber to metformin monotherapy improves glucose tolerance. However, possible synergistic effects of combination therapy (metformin plus fiber) have not been investigated in humans. Moreover, the underlying mechanisms of synergy have yet to be elucidated. The aim of this study is to compare in adolescents with obesity the metabolic effects of metformin and fermentable fibers in combination with those of metformin or fiber alone. We will also determine if therapeutic responses correlate with compositional and functional features of the gut microbiome.

**Methods:**

This is a parallel three-armed, double-blinded, randomized controlled trial. Adolescents (aged 12–18 years) with obesity, insulin resistance (IR), and a family history of type 2 diabetes mellitus (T2DM) will receive either metformin (850 mg p.o. twice/day), fermentable fibers (35 g/day), or a combination of metformin plus fiber for 12 months. Participants will be seen at baseline, 3, 6, and 12 months, with a phone follow-up at 1 and 9 months. Primary and secondary outcomes will be assessed at baseline, 6, and 12 months. The primary outcome is change in IR estimated by homeostatic model assessment of IR; key secondary outcomes include changes in the Matsuda index, oral disposition index, body mass index *z*-score, and fat mass to fat-free mass ratio. To gain mechanistic insight, endpoints that reflect host-microbiota interactions will also be assessed: obesity-related immune, metabolic, and satiety markers; humoral metabolites; and fecal microbiota composition, short-chain fatty acids, and bile acids.

**Discussion:**

This study will compare the potential metabolic benefits of fiber with those of metformin in adolescents with obesity, determine if metformin and fiber act synergistically to improve IR, and elucidate whether the metabolic benefits of metformin and fiber associate with changes in fecal microbiota composition and the output of health-related metabolites. This study will provide insight into the potential role of the gut microbiome as a target for enhancing the therapeutic efficacy of emerging treatments for T2DM prevention.

**Trial registration:**

ClinicalTrials.gov NCT04578652. Registered on 8 October 2020.

**Supplementary Information:**

The online version contains supplementary material available at 10.1186/s13063-021-05060-8.

## Administrative information

The order of the items has been modified to group similar items (see http://www.equator-network.org/reporting-guidelines/spirit-2013-statement-defining-standard-protocol-items-for-clinical-trials/).

**Table:** 

Title {1}	Metformin and fermentable fiber combination therapy in adolescents with severe obesity and insulin resistance: Study protocol for a double-blind randomized controlled trial.
Trial registration {2a and 2b}.	ClinicalTrials.gov Identifier: NCT04578652
Protocol version {3}	Protocol version 1.2 dated 07 October 2020
Funding {4}	This work is funded by The W. Garfield Weston Foundation (without project number)
Author details {5a}	University of Alberta, Edmonton, Canada California State University, California, United States University of Hong Kong, Sandy Bay, Hong Kong University College Cork – National University of Ireland, Ireland Duke University Medical Center, North Carolina, United Stated
Name and contact information for the trial sponsor {5b}	University of Alberta Quality Management in Clinical Research (QMCR) Department Tel.: (780) 292–3649
Role of sponsor {5c}	The University of Alberta and The W. Garfield Weston Foundation will not intervene in any aspect of the trial, including its design, data collection, analysis, or presentation.

## Introduction

### Background and rationale {6a}

Childhood obesity is a major risk factor for the development of insulin resistance (IR) and type 2 diabetes mellitus (T2DM) in youth. Importantly, youth-onset T2DM has been delineated as a more aggressive disorder, characterized by severe IR, insulin hypersecretion, rapid β-cell deterioration, and poor response to standard therapies [1–4]. Although limited or modest success has been reported for lifestyle interventions with behavioral modifications, such as diet and physical activity, they remain the most commonly applied therapies for IR and the underlying obesity in both adults and adolescents [1]. Pharmacotherapy with metformin (MET) has shown to induce modest reductions in body mass index (BMI) and reverse glucose intolerance in adult-onset T2DM [5]. However, for youth-onset T2DM, MET monotherapy has shown a 52% failure rate, while MET plus intensive lifestyle therapy also has shown a failure rate of 47%, as estimated by an uncontrolled intervention trial [1, 4]. Therefore, a clear need exists for the identification of efficacious therapies in adolescents which could be added to lifestyle modifications.

Adolescents with obesity typically present with systemic low-grade inflammation that has been implicated in the development of IR and cardiovascular dysfunction [6]. Obesity has also been associated with an imbalance in the composition and functionality of the gut microbiota [7–9]. However, it is unclear if changes in the gut microbial community contribute to the pathophysiology of adolescent obesity and associated comorbidities [10–12]. Gut microbiota might influence obesity and its dysregulated immunometabolism by facilitating caloric recovery [13], altering intestinal barrier and immune homeostasis [14], promoting the release of incretins and satiety hormones such as glucagon-like peptide 1 (GLP-1) [15], and regulating intestinal and hepatic gluconeogenesis [16]. Research further suggests that the composition of the gut microbiota can alter the risk of developing IR. For instance, the abundance of *Akkermansia muciniphila* has been inversely associated with obesity and IR [17–19]. On the other hand, *Prevotella copri* and *Bacteroides vulgatus* have been positively associated with the biosynthesis of branched chain amino acids (BCAAs), which are linked to the development of IR [20, 21]. Finally, the translocation of microbially derived lipopolysaccharides, termed endotoxemia, has been observed in patients with metabolic syndrome and T2DM [17, 22]. Despite the relationship described before, causal links between IR and the human gut microbiota remain to be established.

Effects of MET on glucose homeostasis are mediated through increases in hepatic insulin sensitivity, intestinal glucose utilization, anorexigenic GLP-1 levels, and reductions in hepatic glucose production [23]. At least some of the effects of MET are thought to be mediated by the gut microbiota. Recently, Wu and colleagues investigated the effect of MET on the composition and metabolic functions of the gut microbiota using a parallel-armed, randomized, placebo-controlled study in adults with newly diagnosed T2DM [24]. On an energy restricted diet, both groups reduced BMI significantly; however, only the MET group demonstrated reductions in fasting blood glucose and hemoglobin A1c (HbA_1c_) after 4 months. MET was found to increase the fecal abundances of *Bifidobacterium adolescentis* and *A. muciniphila*, with both species further shown to utilize MET for growth in vitro. However, HbA_1c_ reductions were only associated with changes in *B. adolescentis*, suggesting a positive link between MET-improved glycemic control and the abundance of *B. adolescentis* [24]. More recently, administration of *A. muciniphila* has been shown to improve insulin sensitivity in adults with obesity and IR [25].

Dietary fibers have been long implicated in the protection from obesity and related comorbidities [26, 27]. As dietary fibers are not digested in the small intestine, they serve as growth substrates for the gut microbiota, producing putatively beneficial metabolites such as short-chain fatty acids (SCFAs) upon degradation [28, 29]. Microbially derived SCFAs have been proposed to enhance intestinal barrier function, downregulate pro-inflammatory immune responses, alter gastrointestinal transit time, reduce hepatic-associated gluconeogenesis, improve insulin sensitivity, and promote satiety independent of GLP-1 [29–31]. Administration of SCFAs in humans has also been shown to improve glucose metabolism, systemic inflammation, and energy homeostasis [30]. In addition, consumption of diets rich in dietary fiber can limit microbial fermentation of proteins and production of potentially detrimental metabolites, such as *p*-cresol, amines, and branched chain fatty acids [29, 32, 33]. Since MET and fermentable fibers have been shown to reduce weight and increase insulin sensitivity through divergent mechanisms of action, we postulate that combination therapy with MET plus fiber will act in concert to increase insulin sensitivity in adolescents with obesity through synergistic effects on the gut microbiome.

Pre-clinical studies in animal models of diabetes confirm that the addition of fermentable fibers, such as type-III resistant starch [34], PolyGlycopleX® (PGX) [35], and konjac mannan oligosaccharides [36], to MET monotherapy enhances glycemic control and delays T2DM progression. While evidence in humans remains limited, a short-term, uncontrolled study in adolescents showed that MET plus fiber (Policaptil Gel Retard®) promoted greater weight loss than MET alone; however, links to the gut microbiome were not elucidated [37]. In adults, the addition of inulin, oat β-glucan, and blueberry-derived polyphenolics to MET monotherapy has been shown to improve both glycemia and the gastrointestinal tolerance of MET after 2 weeks [38]. Furthermore, consumption of either supplemental fiber or a high-fiber diet promoted weight loss and improved HbA_1c_ in adults with T2DM on MET monotherapy [39]. Overall, these findings suggest that separate pathways underlie the effects of MET and fermentable fibers, as fermentable fibers enhanced responses to MET. Thus, gut microbiome-targeted MET plus fiber combination therapies may have potential for enhanced reduction of IR in adolescents with obesity. The aim of this study protocol is to compare the effects of MET and fiber alone and in combination over 12 months on measures of insulin sensitivity and resistance in adolescents at high risk of T2DM.

### Objectives {7}

The primary objective is to compare the efficacy of MET (850 mg p.o. twice/day) versus FIBER (35 g/day supplemental fiber) alone versus combined MET plus FIBER on IR (as estimated by homeostatic model assessment of insulin resistance [HOMA-IR]) in adolescents with obesity, IR, and family history of T2DM.

The secondary objective is to compare the effects of the study therapies alone or in combination on:

1. Changes in the Matsuda, insulinogenic, and oral disposition indices as determined by OGTT
2. Changes in body weight, BMI percentile and *z*-score, and body composition (fat mass, fat-free mass, and fat mass to fat-free mass ratio)
3. Changes in quality of life (QoL) and perceived hunger and satiety
4. Changes in fasting metabolic (glucose, adiponectin, and lipids) and satiety markers (acylated ghrelin, peptide tyrosine tyrosine [PYY], GLP-1, and leptin)
5. Changes is measures of systemic inflammation (C-reactive protein, interleukin 6, and tumor necrosis factor-α [TNF-α]) and intestinal barrier function (lipopolysaccharide-binding protein (LPB) and fecal calprotectin)
6. Changes in gut microbiome composition and functions (fecal microbiota composition, fecal SCFAs and bile acids, and targeted plasma metabolomics [amino acids, branched chain ketoacids, acylcarnitines, ceramides, trimethylamine *N*-oxide, choline, and betaine])

### Trial design {8}

**Fig. 1:**
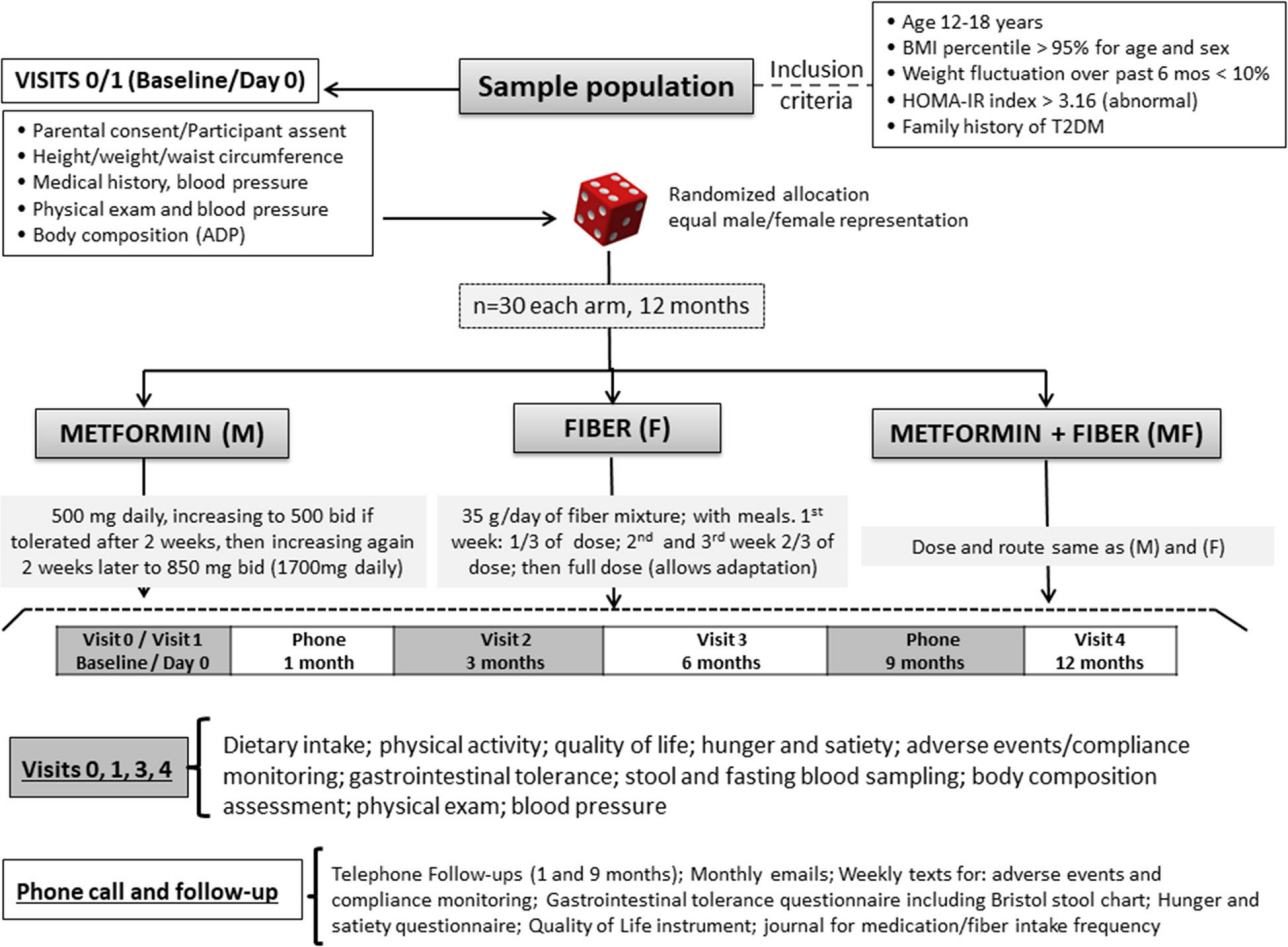
Study design. Participants meeting the eligibility criteria will be randomly allocated to one of three study groups: (1) metformin (850 mg bid), (2) fiber (supplemental fiber 35 g/day) or metformin plus fiber. Participants will be followed up for 12 months with clinical visits every 3 months. Abbreviations: ADP, air displacement plethysmography; BMI, body mass index; HOMA-IR, homeostatic model assessment of insulin resistance; T2DM, type 2 diabetes mellitus

**Fig. 2:**
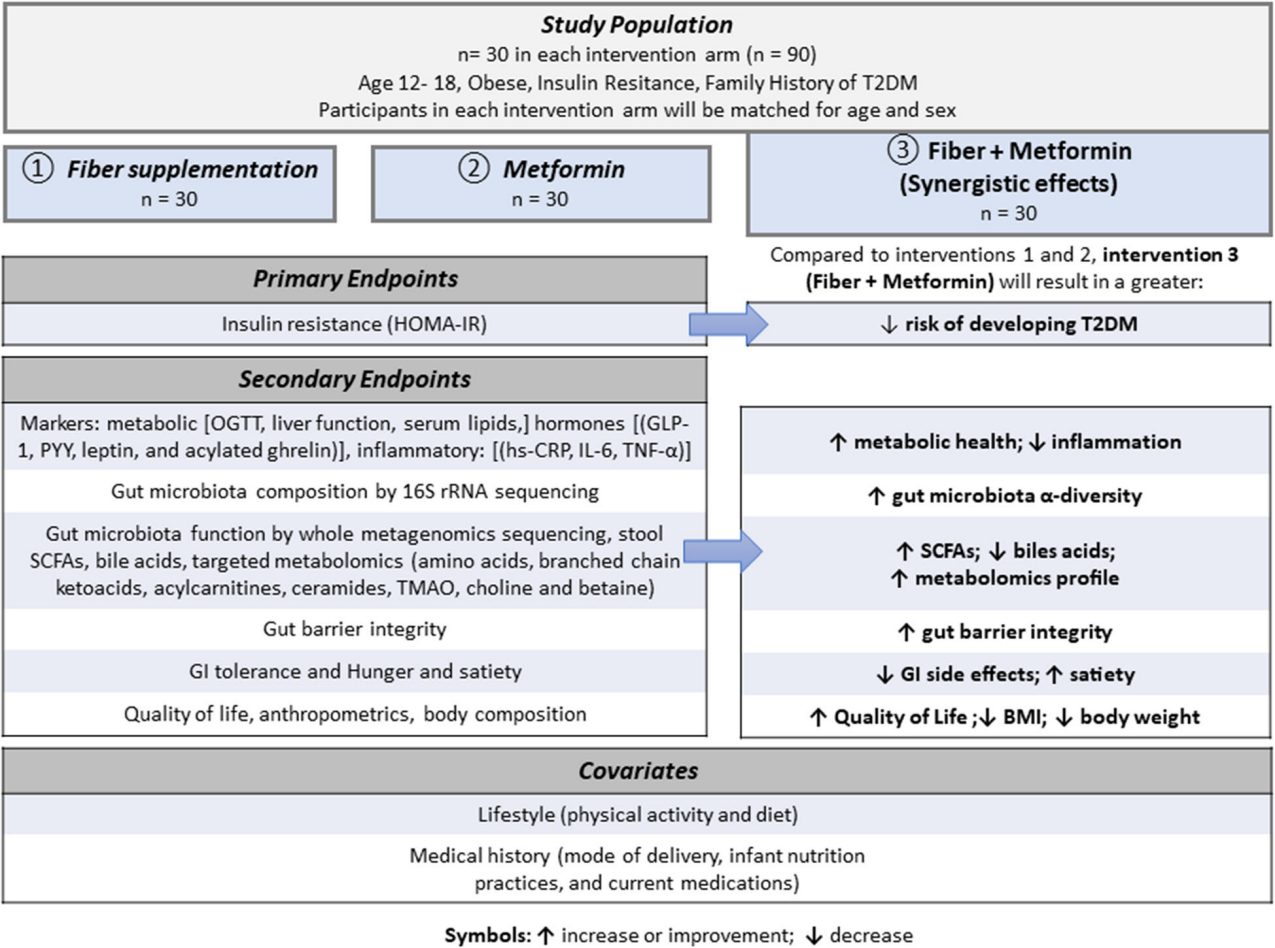
Conceptual design. Changes expected in primary and secondary outcomes in the combination therapy compared to each monotherapy. Symbols: ↑ increase or improvement; ↓ decrease

A single-center, parallel three-armed, double-blinded, 12-month randomized controlled trial with a 1:1:1 allocation ratio. Ninety adolescents (*n* = 30 per arm) with obesity, IR, and family history of T2DM will be enrolled in the trial matched for sex and age (Figs. 1 and 2).

## Methods: participants, interventions and outcomes

### Study setting {9}

Participants will be recruited from both the Pediatric Endocrinology and General Pediatric Clinic at the Stollery Children’s Hospital at the University of Alberta (UofA), and the community based Pediatric Centre for Weight and Health in Edmonton, Canada.

### Eligibility criteria {10}

Inclusion criteria:

1. Age 12–18 years old
2. BMI ≥ 95th percentile for age/sex
3. Total weight fluctuation over past 6 months < 10%
4. HOMA-IR > 3.16
5. Family history of T2DM (first- or second-degree relative)

Exclusion criteria:

1. Current use of insulin or diagnosis of T2DM
2. Systolic or diastolic blood pressure > 99th percentile for age and sex
3. Acute infectious or inflammatory condition over the preceding 1 month; hospitalization > 48 h
4. History of chronic diseases, such as liver, kidney, or inflammatory bowel disease, or neurologic disorders
5. Active malignancy
6. Concomitant use of medication/investigational drug known to affect body weight in the past year
7. Antibiotic use in past 60 days; probiotic and/or prebiotic supplement use in the past 30 days; use of lipid-lowering and anti-inflammatory medication

### Who will take informed consent? {26a}

Written informed consent (from parents/caregivers and participants aged 18 years) and assent (from participants aged 12–17 years) will be obtained from all participants before inclusion by the principal investigator or trained research staff.

### Additional consent provisions for collection and use of participant data and biological specimens {26b}

Participants will be asked to provide Optional Specimen Consent to biobank blood and stool samples for future studies yet to be determined. Participants who do not consent for biobanking can still take part in the rest of the study.

## Interventions

### Explanation for the choice of comparators {6b}

We postulate that the therapeutic effects of both MET and fermentable fibers are mediated, in part, through diverging effects on the gut microbiome, and that MET plus fiber combination therapy will act synergistically to improve glucose tolerance in adolescents with obesity and IR. By combining MET with fiber, we anticipate that the improvement in IR and BMI will be significantly higher when compared to each monotherapy. A control group without treatment is not included, since a no treatment arm would be unethical for adolescents with obesity and IR.

### Intervention description {11a}

Participants will be randomly assigned to one of three study arms:

1. MET arm: MET (850 mg p.o. twice/day—standard of care) plus fiber placebo daily
2. FIBER arm: fiber supplementation (35 g/day fiber) plus MET placebo twice/day
3. MET + FIBER arm: MET (850 mg p.o. twice/day) plus fiber supplementation (35 g/day fiber)

### Metformin administration and rationale

Participants in the MET group will initially receive 500 mg daily, increasing to 500 mg twice/day if tolerated after 2 weeks (those who do not tolerate will be withdrawn from the study), and then increasing after an additional 2 weeks to 850 mg twice/day (1700 mg daily). The MET or placebo (microcrystalline cellulose [MCC] powder) capsules will be taken with meals along with a multivitamin containing B12, in order to prevent a potential MET-associated vitamin B12 deficiency [40]. A dose of 850 mg twice/day was chosen based on studies demonstrating decreases in BMI and IR at these doses [41, 42]. Although side-effects of MET are generally minor, the dosage will be titrated to avoid mild, self-limited side-effects (i.e., abdominal pain, flatulence, bloating, nausea, and diarrhea). Although lactic acidosis, hypoglycemia, and other serious side-effects are rare, side-effects will be routinely monitored throughout the trial by the principal investigator or a research coordinator.

### Fiber administration and rationale

Our supplemental fiber mixture (35 g/day of total fiber) will be composed of fermentable non-viscous (6 g oligofructose + 12 g resistant maltodextrin + 12 g acacia gum) and viscous (5 g PGX) fibers. Dosages of individual fibers were determined based on clinical evidence for effective dose and known tolerability data (Additional file 1). In addition, we and others suggest that ~ 50 g/day of fiber (35 g/day supplemental + ~ 15 g/day from diet) may be required for attaining reliable physiological benefits linked to fiber [29, 43]. This conclusion is supported by recent findings that 35 g/day of fermentable fiber maximized the health-relevant shifts in both bacterial taxa and fecal SCFAs [33]. Previous pediatric dietary fiber interventions of similar dosage report no tolerance concerns [44, 45]. For instance, Zhang et al. provided children with obesity around 50 g/day of fiber without any concerns of tolerance [45]. While it is expected that 35 g/day of fermentable fiber will be tolerated, gastrointestinal symptoms will be monitored throughout the trial.

To allow time for gastrointestinal adaptation, participants will be instructed to use ^1^/_3_ of the total daily fiber dose (or placebo) during the first week of treatment; then ^2^_/3_ of the dose for the second and third weeks; and then the full dose thereafter. This dose escalation is suggested to improve tolerance as adaption over time has been previously described for such fiber supplements [46, 47]. The fiber treatment (or placebo) will be provided as a powder to be added by the participant to water or sugar-free beverages and consumed prior to meals. This method of consumption is easy to incorporate and ensures enough water intake is spread throughout the day to improve gastrointestinal tolerance. Alternatively, participants will be allowed to mix the fiber, or placebo, with foods (e.g., added to cereals, soups, and yogurt), if preferred, to allow flexibility and maximize compliance, which is important due to the duration of the intervention. The fiber placebo will consist of MCC, a non-fermentable fiber with no effect on the gut microbiota [48].

### Criteria for discontinuing or modifying allocated interventions {11b}

Participants will be withdrawn from the study if the participant (1) withdraws consent, (2) becomes pregnant, (3) does not tolerate either MET or dietary fiber interventions, (4) requires antibiotic therapy within the first 6 months of the trial, (5) has an HbA1c > 8%, or (6) the participant, in the opinion of the investigator, is not clinically able to continue to follow the investigative intervention (e.g., need of initiating another specific medical intervention such as insulin). Participants will be free to withdraw consent at any time without prejudice to current or future medical care. When a participant expresses his/her wishes to withdraw from the study, he/she will receive instructions to complete an “end of study” visit, which will also be voluntary. Data collected up to the time of withdrawal will remain in the trial database and be included in data analysis.

### Strategies to improve adherence to interventions {11c}

We will document and reinforce adherence during each study visit. Participants will complete a dosing journal (self-documentation) and return unused products; research staff will review the journal and number of remaining pills or sachets containing fiber or placebo to document adherence. Additionally, participants will be contacted regularly by email and text messages to reinforce adherence.

### Relevant concomitant care permitted or prohibited during the trial {11d}

Hypoglycemic drugs, insulin, and medications known to affect body weight will not be allowed during the trial. If a participant must use any of these medications, he/she will be withdrawn from the study. The principal investigator will determine acceptability of any other concomitant medication. Adolescents in this study will continue to receive conventional lifestyle management advice as per routine clinical practice at their clinics.

### Provisions for post-trial care {30}

There is no specified ancillary or post-trial care for participants in this trial. However, it is expected that the results of this study will guide clinical care of children at high risk of T2DM after completion of the trial. If a participant becomes ill or injured as a result of being in this study, he/she will receive necessary medical treatment, at no additional cost to the participant.

### Outcomes {12}

The primary outcome of this study is a change in IR, as estimated by HOMA-IR, between baseline, 6, and 12 months. The secondary outcomes of this study include changes between baseline, 6, and 12 months in (1) the Matsuda, insulinogenic, and oral disposition indices as determined by OGTT; (2) weight, BMI percentile and *z*-score, and body composition (fat mass, fat-free mass, and ratio of fat mass to fat-free mass); (3) QoL and perceived hunger and satiety; (4) fasting metabolic (glucose, adiponectin, lipids, and OGTT) and satiety markers (acylated ghrelin, PYY, GLP-1, and leptin); (5) measures of systemic inflammation (C-reactive protein, interleukin 6, and TNF-α) and gut barrier integrity (lipopolysaccharide-binding protein [LPB] and fecal calprotectin); and (6) gut microbiome composition and functions (fecal microbiome composition, fecal SCFAs and bile acids, and targeted plasma metabolomics [amino acids, branched chain ketoacids, acylcarnitines, ceramides, trimethylamine *N*-oxide, choline, and betaine]).

### Participant timeline {13}

#### Sample size {14}

Our primary outcome is IR as estimated by HOMA-IR. Based on a recent study where the effect size of MET on HOMA-IR among pubertal participants was 0.75 [49], we estimated a sample size of 29 per arm, which will give us 90% power to find any significant post-intervention difference between arms at 5% significance level. Additionally, a percentage of change and 95% confidence interval for HOMA-IR after PGX from Pal et al. [50] resulted in an effect size of 0.86, rendering a sample size of 26 per arm. We expect a greater response in the combination therapy; for this reason and considering 26 per arm as feasible, and 15% attrition rate during follow-up, we estimated a total of 90 participants over the 3 arms.

### Recruitment {15}

Participants will be recruited over 24 months. Potential participants will be identified by clinicians that are aware of the study, the principal investigator, or research staff who will review the clinical charts of patients attending the Pediatric Endocrinology Clinic, General Pediatric Clinic, and the Pediatric Centre for Weight and Health. Once a potential participant has been identified, a clinician or the principal investigator will approach the potential participant and their parents, provided information about the study, and ask the patient if his/her name can be passed onto a research team member to be contacted about the study. If the potential participant and their parents provide written consent to release contact information to researcher, the research team member will contact them to provide additional information about the study, answer questions or concerns, complete a screening process, and if applicable, arrange a potential study visit where written inform consent will be obtained. The informed consent process will be conducted in person by a qualified research team member.

In the case that a research staff member identifies a potential participant, the study coordinator will approach the attending physician and review this information. If the attending physician agrees, he/she will ask the patient if they might be interested in learning more about the research study. If the patient agrees, a member of the research team will be called to share the information and invite the patient to participate.

## Assignment of interventions: allocation

### Sequence generation {16a}

**Fig. 3:**
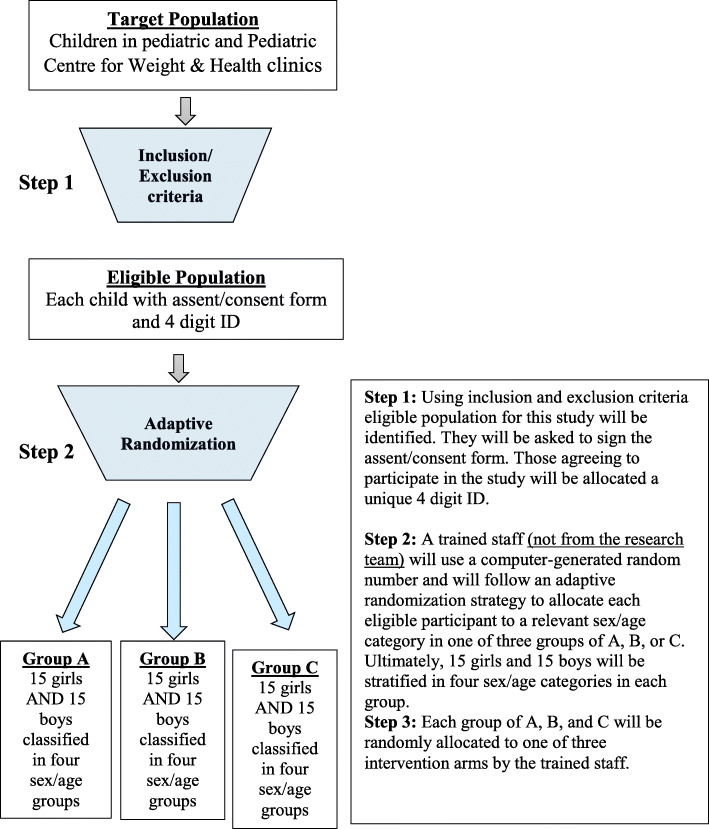
Study population recruitment and randomization strategy

After having provided written informed consent/assent, participants will undergo a baseline assessment and be randomized to one of the three intervention groups A, B, and C (i.e. MET, FIBER, MET + FIBER) via computer-generated numbers and stratified by age and sex, using the adaptive (dynamic) randomization (Step 2, Fig. 3). Each group will be also randomly allocated to one of three intervention arms of MET, FIBER, and MET + FIBER (Step 3, Fig. 3). Staff who completed Steps 2 and 3 will be different and will be blinded to the other step. Stratification will be based on four sex/age categories of male 12–15 years old, female 12–15 years old, male 16–18 years old, and female 16–18 years old.

### Concealment mechanism {16b}

The arm allocations will be concealed by keeping this information restricted by the statistician generating the randomizations codes.

### Implementation {16c}

A statistician will generate the randomization codes and an unblinded research team member will package and label the investigational fibers and placebo. These research personnel will not be involved in any other study procedures/assessments. Different research staff will enroll and randomize participants.

## Assignment of interventions: blinding

### Who will be blinded {17a}

Both participants and research team will be blinded to the type of intervention allocated to each arm until the end of the study. The group not on MET (fiber alone) will receive a placebo pill (MCC). The group not on fiber will also receive a placebo (MCC) in identical sachets; this double-blind strategy will reduce risk of bias. Both fiber and MET have a similar side-effect profile, which will support the blinding procedures.

### Procedure for unblinding if needed {17b}

In case of an emergency in which knowledge of the treatment assignment is deemed essential by the participant’s care, the code could be opened. The person in charge of keeping these codes secure will open the code for that specific participant and will inform only the treating physician, keeping the code concealed from research personnel. Any unblinding will be approved by the principal investigator.

## Data collection and management

### Plans for assessment and collection of outcomes {18a}

Study visits will be conducted at the Human Nutrition Research Unit, a state-of-the-art facility supporting leading nutrition intervention research at the UofA. Participants will be seen at baseline (visits 0 and 1), 3, 6, and 12 months, and a phone follow-up will be completed at 1 and 9 months (Table 1). The 12-month duration of the intervention will assess both short- and longer-term effects of the combined FIBER plus MET intervention. At visit 0, and after obtaining informed consent/assent, baseline assessments will be conducted, including demographics, medical history, physical exam, sexual maturation (Tanner stage), anthropometrics, and body composition; in addition, participants will complete QoL, gastrointestinal tolerance, and 7-day physical activity questionnaires. During this visit, research staff will provide to the participants a 3-day food record (to assess dietary intake), Satiety Labeled Intensity Magnitude (SLIM) and bowel habit (Bristol stool chart) questionnaires, and stool sample collection kits to be completed at home and brought to visit 1 (day 0); this visit will occur within the following 10 days after visit 0. At visit 1, fasting blood samples will be withdrawn, and an OGTT will be completed. Once baseline assessments are completed, participants will be randomly assigned to one of the three study groups and the respective study intervention will be delivered.

**Table 1:**
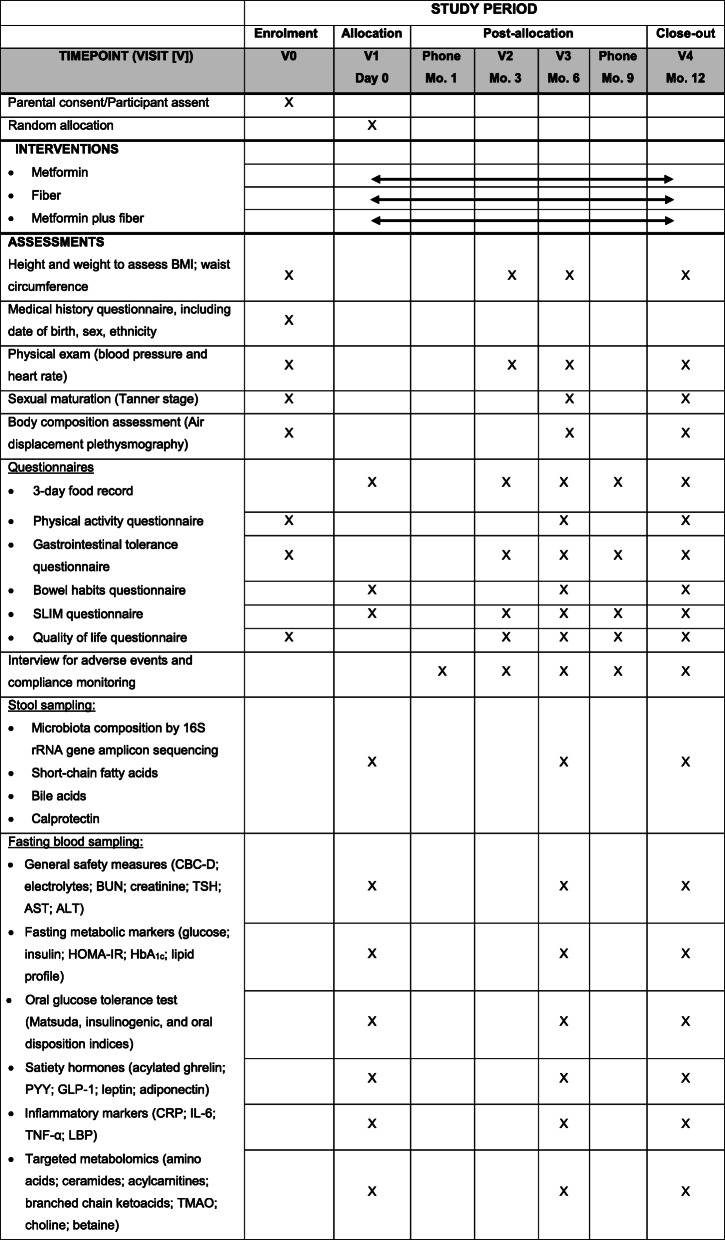
Time schedule of enrolment, intervention, assessments, and study visits for participants

Abbreviations: *ALT* alanine aminotransferase, *AST* aspartate transaminase, *BUN* blood urea nitrogen, *CBC-D* complete blood count with differential, *GLP-1* glucagon-like peptide 1, *HbA*_*1c*_ hemoglobin A1C, *HOMA-IR* homeostatic model assessment of insulin resistance, *CRP* C-reactive protein, *IL-6* Interleukin 6, *LBP* lipopolysaccharide-binding protein, *PYY* peptide tyrosine tyrosine, *SLIM* satiety labeled intensity magnitude, *TMAO* trimethylamine *N*-oxide, *TNF-α* tumor necrosis factor alpha, *TSH* thyroid-stimulating hormone

Initial assessments, including primary and secondary outcomes, will be repeated at 6- and 12-month follow-up visits. At 1 month, participants will be contacted over the phone to monitor adverse events and compliance. During the 3-month visit, participants will undergo a physical exam and anthropometric assessments and will complete QoL, gastrointestinal tolerance, and SLIM questionnaires, as well as a 3-day food record. At 9 months (phone follow-up), only questionnaires and a 3-day food record will be completed; research staff will assist in completing these questionnaires over the phone when needed (Table 1).

### Demographics and clinical assessment

Date of birth and ethnicity will be collected. A medical history (mode of delivery and infant nutrition practices [breastfeeding or formula], and current medications) will be completed, a physical exam (blood pressure and heart rate using an automated blood pressure monitor) will be conducted, and sexual maturation will be self-assessed (by children assisted by their parents) using the Tanner Stage scale [51].

### Anthropometrics and body composition

After participants void their bladders, body weight and height will be measured and used to calculate BMI percentile and *z*-score (WHO Anthroplus software, Geneva, Switzerland). Waist circumference will be measured in narrowest site between the xiphoid process and iliac crest [52]. Waist circumference *z*-score will be calculated using the Anthropometric Calculator for normal children 5–19 years of age based on the World Health Organization Growth Charts for North America Children [53]. Body density using air displacement plethysmography (Bod Pod® 1SB-060 M, Life Measurement Instruments, CA, USA) [54] will be assessed to estimate fat mass, fat-free mass, and the ratio of fat mass to fat-free mass, according to the manufacturer’s instructions [55].

### Study questionnaires

A 3-day food record (2 weekdays/1 weekend day) will be completed every 3 months to monitor dietary intake and diet quality throughout the study. Self-reported physical activity levels will be assessed every 6 months using the physical activity questionnaire for older children (≤ 14 years) [56] and for adolescents (> 14 years) [57]. QoL will be measured every 3 months using the Peds QL 4.0 instrument [58]. The validated SLIM scale will be used to assess perceived hunger and satiety (fasting, before dinner, and 1 and 2 h after dinner) every 3 months. This scale is a 100-mm visual analog scale anchored by “greatest imaginable hunger” and “greatest imaginable fullness”, with “neither hungry nor full” in the center. Participants will place a mark on the scale corresponding to their sensation of hunger or fullness [59]. To evaluate the gastrointestinal tolerance of the interventions, a previously described gastrointestinal tolerance questionnaire will be used every 3 months [33]. This questionnaire assesses the presence and severity of six symptoms over the previous 7 days, including nausea, gastrointestinal rumblings, abdominal pain, bloating, flatulence, and diarrhea. Participants will rate each symptom as “did not experience,” “no more than usual,” “somewhat more than usual,” or “much more than usual” [33]. Bowel habits will also be recorded over 4 days every 6 months by using a bowel habits diary; in this diary, participants will record bowel movement frequency and fecal consistency rated on a scale of 1 (hard) to 5 (watery) using the Bristol Stool Scale for children [60].

### Assessment of insulin resistance, metabolites, hormones, and intestinal barrier function

As the primary endpoint, HOMA-IR will be calculated as fasting glucose (mmol/L) × fasting insulin (μIU/mL)/22 [61, 62]. HOMA-IR has been shown to be reproducible and correlate with more invasive tests of insulin sensitivity [63, 64]. An OGTT will also be completed and the Matsuda (whole body insulin sensitivity index) [65], insulinogenic [66], and oral disposition [67] indices well be calculated. The OGTT has been validated in multiple clinical and research settings and reflects the efficiency of the body to dispose of glucose after an oral load; it is commonly used to diagnose glucose intolerance and diabetes [68–70]. The participants at risk for T2DM will already require regular screening using OGTT every ~ 6 months; thus, utilizing this gold standard for the study will not create extra patient burden. Participants will ingest 1.75 g/kg (75 g maximum) glucose; blood samples for glucose and insulin will be obtained at 0, 30, 60, and 120 min.

Fasting (12 h) plasma and serum samples will be collected and stored at − 80 °C until further analysis. These analyses include the measure of (1) general safety measures (CBC-D, electrolytes, blood urea nitrogen, creatine, thyroid-stimulating hormone, aspartate transaminase, and alanine aminotransferase), (2) satiety markers (acylated ghrelin, PYY, GLP-1, and leptin), (3) metabolic markers (glucose, insulin, HbA_1c_, total and high-molecular-weight adiponectin, total cholesterol, high-density lipoprotein cholesterol, low-density lipoprotein cholesterol, and triglycerides), (4) inflammatory markers (C-reactive protein, interleukin 6, TNF-α, and lipopolysaccharide-binding protein [LBP; a measure of intestinal barrier function]), and (5) targeted metabolites measured by liquid chromatography–tandem mass spectrometry (amino acids, branched chain ketoacids, acylcarnitines, ceramides, trimethylamine *N*-oxide, choline, and betaine). Protease inhibitors will be added when required for the quantification of hormones.

Fecal samples will also be used to measure calprotectin levels (as a measure of intestinal barrier function) using an enzyme-linked immunosorbent assay according to the manufacturer’s protocol.

### Fecal microbiome analyses

Once collected from participants, stool samples will be immediately processed, aliquoted, and stored at − 80 °C until further analysis. DNA will be extracted from the fecal homogenates using the QIAamp DNA Stool Mini Kit (Qiagen, Valencia, CA, USA) with the addition of a mechanical lysis step, as recently described by Costea et al. [71]. Fecal microbiota composition will be characterized by 16S rRNA gene amplicon sequencing using MiSeq Illumina technology (pair-end) as previously described [33]. Quality-controlled reads will be analyzed using (1) taxonomic-based approaches (GAST and the Ribosomal Database Project Multi Classifier tool) and (2) non-taxonomic-based clustering algorithms for operational taxonomic unit (OTU) determination with the UPARSE pipeline. α-diversity (Shannon, Simpson, and observed OTUs) and β-diversity indices (Bray-Curtis and binary Jaccard) will be calculated in QIIME2 [72] and R (VEGAN package) [73]. To assess metabolic functions of the gut microbiota, fecal SCFAs will also be analyzed by gas chromatography (Varian, CA, USA) [74] and bile acids using liquid chromatography–mass spectrometry [75, 76].

### Plans to promote participant retention and complete follow-up {18b}

When a participant expresses his/her wishes to withdraw from the study, he/she will be asked to complete an “end of study” visit. Data collected up to the time of withdrawal will remain in the trial database and be included in data analysis, unless otherwise indicated by the participant.

### Data management {19}

Study charts will be stored within a secure cabinet at site. All research data will be captured and managed using Research Electronic Data Capture (REDCap) [77] hosted at the Faculty of Medicine and Dentistry at the UofA. To ensure data quality, the database will be designed with branching logic, data validation, and range checks for data values, where possible. Research data and study documentation will be retained for a period of 25 years.

### Confidentiality {27}

No directly identifying information will be entered in the REDCap system, and participants will be identified by a unique participant study number (code) on the case report forms. Personnel entering research data into REDCap will have a personal username and password after access has been granted by the REDCap administrator. This password will be required to be changed periodically. Other study-related documents containing direct identifiers (e.g., signed consent form) will be stored in a locked filing cabinet in a secure office at site. All computer files related to this study (e.g., master list and data set) will be encrypted and password protected. Participants will be informed during the consent inform process that the coded research data and original medical records may be inspected by UofA auditors, members of the Research Ethics Board, and Health Canada, for regulatory and monitoring purposes.

### Plans for collection, laboratory evaluation, and storage of biological specimens for genetic or molecular analysis in this trial/future use {33}

Stool and blood samples will be collected at site from the participant and processed. Then, aliquoted stool, serum, and plasma will be stored at − 80 °C in the freezer located at site until further analysis for the same purpose of the study objectives. Additional samples from participants who provided Optional Specimen Consent for biobanking and genetic testing will be kept up to 15 years, until they are used up for future studies, yet to be determined, or destroyed.

### Statistical methods

#### Statistical methods for primary and secondary outcomes {20a}

Descriptive statistics will summarize all study variables. Prior to analysis, numerical variables with skewed distribution will be transformed (e.g., log_2_ or cubed root) or a comparable nonparametric test will be used. Between-group comparison of primary and secondary outcomes will be performed at baseline and two consecutive time points independently using unpaired *t*-tests (comparing two groups) or analysis of variance (three groups) and chi-square tests for numerical and categorical data, respectively. Impact of the three interventions on IR (HOMA-IR, primary endpoint) over time will be compared using linear mixed models, after adjusting for relevant confounders, including pubertal stage if any statistically significant difference is observed between treatment groups at baseline. OTU relative abundance will be compared between treatment groups. Statistical analyses for gut microbiota community composition will include principal coordinates analysis and canonical correlation. Linear discriminant analysis Effect Size [78] and multivariate association with linear models will be used to identify specific OTUs that differentiate the treatment groups. Mediation modeling will be employed to provide further insights on possible casual pathways to explore whether the gut microbiota may play a causal or mediation role in the physiological effects detected [79]. Treatment responders versus non-responders will also be evaluated. We will characterize the ecological differences prior to the intervention, and then the individualized response to the intervention in order to assess the role of the microbiome in the host’s metabolic response to the intervention. For this analysis, we will apply a machine learning approach called partition around medoids clustering [80]. Separate models will be estimated for secondary endpoints, which are exploratory, but nevertheless important. Intervention types and times will be included as fixed effects in linear mixed models. Pearson or Spearman correlation coefficients will be computed between changes in gut microbiome composition/function and insulin resistance, hormones, metabolites, and inflammatory markers.

#### Interim analyses {21b}

No interim analyses will be completed.

#### Methods for additional analyses (e.g., subgroup analyses) {20b}

We plan to do a subgroup analysis for age and sex differences within each arm.

#### Methods in analysis to handle protocol non-adherence and any statistical methods to handle missing data {20c}

We will undertake an intention-to-treat analysis to assess intervention effect.

Multiple imputation approach or a sensitivity analysis will be followed to address missing data.

#### Plans to give access to the full protocol, participant-level data, and statistical code {31c}

There are no plans for granting public access of the full protocol, participant-level dataset, or statistical code.

### Oversight and monitoring

#### Composition of the coordinating center and trial steering committee {5d}

The study will be conducted under the leadership of a Steering Committee, which will be responsible for scientific and operational guidance to the overall protocol. The Steering Committee will meet monthly in the initial phases, then quarterly throughout the project remainder. A Data Safety Monitoring Board made of experts in pediatric obesity and T2DM will provide oversight and will be assembled on an ad hoc basis.

#### Composition of the data monitoring committee, its role and reporting structure {21a}

Site initiation, ongoing monitoring, and study close out will be performed by the Quality Management in Clinical Research Group within the UofA (www.qmcr.ualberta.ca/). Interim monitoring visits will be conducted to ensure compliance with Good Clinical Practices; the study is conducted according to site specific standard operating procedures, the protocol, and regulatory guidelines.

#### Adverse event reporting and harms {22}

All adverse events will be tracked in an adverse event log and classified following the Guidance Document for Clinical Trial Sponsors: Clinical Trial Applications [81], according to their severity (serious, non-serious), expectedness (expected, unexpected), and relatedness to the study intervention (related, possible related, unrelated). All serious adverse events will be collected in the participant case report forms. All serious, treatment-related adverse events will be reported to Health Canada and the UofA Human Research Ethics Board (HREB). All serious adverse events will be followed until resolution (for those that resolve before the end of the study), or for 1 month after the end of the study unless the investigator determines that additional follow-up is necessary.

#### Frequency and plans for auditing trial conduct {23}

There are no plans for auditing trial conduct beyond the interim monitoring visits conducted by the Quality Management in Clinical Research Group.

#### Plans for communicating important protocol amendments to relevant parties (e.g., trial participants, ethical committees) {25}

All protocol amendments will be submitted to the UofA-HREB for approval before implementation, unless the amendment is necessary to eliminate an immediate hazard to the trial participants. In this case, the necessary action will be taken first, with the relevant protocol amendment following shortly thereafter. Investigators will be notified once the amendment has been approved by the UofA-HREB.

#### Dissemination plans {31a}

Findings from this trial will be disseminated at local, national, and international academic and professional conferences. It is expected that the study results will be published in scientific peer-reviewed journals.

## Discussion

This study will compare the metabolic benefits of fiber with those of metformin in adolescents with obesity, determine if metformin and fiber act synergistically to improve IR, and elucidate whether the metabolic benefits of metformin and fiber associate with changes in fecal microbiota composition and the output of health-relevant metabolites. The study will thereby assess the relationship between therapeutic intervention(s) and putative mechanisms that are hypothesized to underlie the clinical effects. Overall, we predict that the combination of MET and fiber will have a synergistic effect, being more effective than MET or fiber alone at 12 months in improving IR and BMI in the adolescents with obesity. We further predict that these metabolic benefits will be associated with changes in gut microbial composition and metabolic functions (assessed by 16S rRNA gene amplicon sequencing, fecal SCFAs and bile acids, and targeted plasma metabolomics) and measures of systemic inflammation and intestinal barrier function (assessed by plasma cytokine, LBP, and fecal calprotectin). If successful, our proposed combination therapy may help to interrupt the cycle of weight gain and IR, thereby reducing the risk for developing T2DM, a worldwide public health concern, in adolescence and adulthood.

To potentially maximize the number of responders to the dietary fiber intervention, a combination of fermentable non-viscous (oligofructose, resistant maltodextrin, acacia gum) and viscous (PGX) fibers will be used in this study, providing a complex and diverse array of substrates to the gut microbiota [29, 82]. The colonic microbiota exists as part of an ecosystem, where substantial cross-feeding occurs; metabolites produced by one bacterium are used as a substrate by another bacterium, and specific key-stone species or guilds are required to degrade a substrate [28, 29]. Therefore, a variety of substrates are likely needed to enhance and diversify microbial responses aimed at improving host metabolism. This concept is supported by in vitro evidence showing that a mixture of fibers is better than a single fiber at promoting bacterial diversity [83], which is generally lower in adolescents with obesity [7, 9]. In human trials involving fermentable fibers, a high degree of between-study heterogeneity has been reported in the clinical response to fiber [84, 85]. These inconsistencies might stem from the extensive interindividual differences in gut microbial configurations at baseline and in response to dietary fiber supplementation [48, 84, 86]. This concept is highlighted by the recent work of Hjorth and colleagues, where individuals with a higher *Prevotella*-to-*Bacteroides* ratio at baseline lost significantly more body weight and body fat on a fiber-rich diet than individuals with a lower ratio [87]. Thus, subjects with obesity and an imbalance in the composition and functionality of the gut microbiota may not possess the microbes necessary to utilize and benefit from a single-fiber supplement. Therefore, providing a mixture of fiber structures, as opposed to a single fiber, could potentially induce more reliable metabolic effects in individuals with obesity and IR.

By employing targeted fecal and plasma metabolomics with fecal microbiota sequencing, we can identify changes in metabolite production that correlate with modifications of the gut microbiota. This systematic analysis of the gut microbial community may result in the identification of baseline microbiota configurations or metabolites that predict effects of MET and/or fermentable fibers and could, therefore, be used to enhance clinical responses with personalized therapies. Such novel biomarkers might also be relevant for predicting the future risk of developing metabolic abnormalities associated with childhood obesity. Our novel approach will inform the development of future microbiome-targeted pharmaceutical and prebiotic therapies, including the possible implementation of long-term, multicenter microbiome-targeted intervention studies aimed at improving health outcomes in childhood obesity. Finally, the goal of the research team is to provide a basis for using the gut microbiome as a window to improve the assessment and treatment of metabolic abnormalities in children with obesity.

While the high dietary fiber dose and 12-month intervention period are considered strengths of study, and potentially necessary for attaining reliable and sustained health benefits linked to fiber [85], both variables may impact protocol adherence and attrition throughout the intervention. To partially mitigate this, regular clinic visits, phone calls, emails, and text messages will occur to ensure the participants’ engagement and encourage protocol adherence. We will also monitor adherence through self-documentation (dosing journal) and collecting all unused study products. Another study limitation is that features of the participant’s lifestyle (i.e., diet and physical activity) remain uncontrolled. While this “real life” approach greatly improves the generalizability of study findings, as is often the case, intentional or unintentional lifestyle changes can occur that independently improve IR and BMI. To minimize these effects, participants will be encouraged to follow the study protocol without intentional lifestyle changes. Additionally, 3-day food records and physical activity questionnaires will be completed at baseline and throughout the dietary intervention to establish deviations from baseline, which will be incorporated and controlled through statistical analysis.

In summary, this study will determine the efficacy of MET and fermentable fibers alone or in combination on metabolic control, while also determining if the effects are related to individual differences in microbiome composition and functions. Thus, this study will demonstrate whether the gut microbiome represents a promising target for enhancing therapeutic efficacy and for further preventing T2DM in at risk adolescents. The results of this study may be integrated into clinical practice guidelines for the prevention of youth-onset T2DM and also aid in the development of novel microbiota-targeted therapies for adolescents with obesity and associated metabolic comorbidities.

### Trial status

The protocol published herein is version 1.2 dated 07 October 2020. The trial has not yet started recruitment. Estimated start date of recruitment is April 1, 2021. Estimated end date of recruitment is March 1, 2023.

## Supplementary Information

**Additional file 1.** Tolerance data of fibers with limited gastrointestinal side-effects chosen for the fiber intervention trial. Table presenting data of fibers with limited gastrointestinal side-effects.

## Abbreviations

BMI: Body mass index
GLP-1: Glucagon-like peptide 1
HbA_1c_: Hemoglobin A1c
HOMA-IR: Homeostatic model assessment of insulin resistance
IR: Insulin resistance
LBP: Lipopolysaccharide-binding protein
MET: Metformin
OGTT: Oral glucose tolerance test
OTU: Operational taxonomic unit
PGX: PolyGlycopleX
P.O.: By mouth
PYY: Peptide tyrosine tyrosine
SCFA: Short-chain fatty acid
SLIM: Satiety Labeled Intensity Magnitude
T2DM: Type 2 diabetes mellitus
TNF-α: Tumor necrosis factor-α
UofA-HREB: University of Alberta Human Research Ethics Board
