# Supplementary material for: Efficacy of metformin and fermentable fiber combination therapy in adolescents with severe obesity and insulin resistance: study protocol for a double-blind randomized controlled trial
Source: Trials. 2021 Feb 17;22:148. doi: 10.1186/s13063-021-05060-8 (PMC7890810; doi:10.1186/s13063-021-05060-8)
Supplement: Supplementary file 1 — Additional file 1. Tolerance data of fibers with limited gastrointestinal side-effects chosen for the fiber intervention trial. Table presenting data of fibers with limited gastrointestinal side-effects. [file 13063_2021_5060_MOESM1_ESM.pdf]

Supplemental File 1: Tolerance data of fibers with limited gastrointestinal side-effects chosen for the fiber intervention trial

**Reference**	**Population** Age, years (y) (mean ± standard deviation)	**Type of fiber**	**Dose**	**Tolerance outcomes Summary**
Calame et al., (2011)	Adults;<br><br>Study I: 36.8 ± 16.9<br>Study II: 36.6 ± 12.7	Acacia Gum	5, 10, or 40 g/d	No reported GI discomfort
Babiker et al., (2018)	Adults;<br>50.09 ± 9.3	Acacia Gum	30 g/d	Minor side effects during the first week of the intervention (viscous sensation (32%), diarrhea (11%), nausea (8%), and abdominal bloating (6%). Symptoms subsided within the second week of intake, with only the discomfort of viscous sensation continuing
Babiker et al., (2012)	Adults;<br>Study group:<br>19.37 ± 1.97<br><br>Control group:<br>19.53 ± 2.25	Acacia Gum	30 g/d	Side effects were mild, experienced only in the first week and then resolved. They included unfavorable viscous sensation in the mouth (100%), early morning nausea (81.7%), mild diarrhea (90%) and bloating abdomen (15%).
Cherbut et al., (2003)	Adults;<br>Study I: 22-33 y<br>Study II: 22-38 y	Acacia Gum	10-70 g/d	No difference in tolerance ≤30 g/d. Above this dose, the main complaint was excessive flatulence. However, the mean degree of severity remained mild (<1), even at doses >50 g/d. Other intestinal events were rarely reported.
Weber et al., (2014)	Children;<br>Fiber group: 8.5 ± 1.8<br><br>Control group:<br>7.7 ± 2.4	10.5% Oligofructose, 12.5% Inulin, 24% Acacia Gum, 9% Resistant Starch, 33% Soy Polysaccharide, 12% Cellulose	3.8 and 7.6 g/d (based on participant weight; 3.8 g/d when <18 kg, 7.6 g/d when >18 kg)	Adverse events were not observed for either group, and the products were well tolerated.
Pedersen et al., (2013)	Adults;<br>25.0 ± 1.2	Oligofructose	15, 25, 35, 45, and 55 g/d	All doses were well tolerated with no apparent dose–response effect on GI symptom or general wellbeing scores.
Holscher et al., (2014)	Adults;<br>27.1 ± 4.1	Oligofructose	5 and 7.5 g/d	≤7.5 g/day led to minimal GI upset, with no change in diarrhea, and improved laxation

Liber et al., (2014)	Children;<br>12. 3 ± 2.9	Oligofructose	8 g/d for children 7–11 y<br>15 g/d for children 12–18 y	Adverse effects measured including abdominal pain, flatulence, diarrhoea/loose stool, borborygmi, nausea , and heartburn, did not differ statistically from placebo.
Nicolucci et al., (2017)	Children;<br><br>Prebiotics group:<br>10.4 ± 0.3<br><br>Placebo group:<br>10.2 ± 0.4	Oligofructose - enriched inulin	8 g/d	No gastrointestinal side effects were experienced by 70% of the prebiotic group and 61% of the placebo.<br>A mild ↑ in flatulence and bloating was experienced by 25% and 28% of subjects in prebiotic and placebo, respectively.<br>A moderate ↑ in flatulence and bloating was reported by 5% and 11% of subjects in prebiotic and placebo, respectively.<br>No reports of severe effects in either group.
Pasman et al., (2006)	Adults;<br>Glucidex® group:<br>33.8±10.1<br><br>30g Nutriose® FB group: 35.7±7.6<br><br>45g Nutriose® FB group: 34.4±7.4	Resistant Maltodextrin	30 or 45 g/d	Both doses were very well tolerated and GI complaints did not differ greatly from the placebo treatment. Some habituation and adaptation of the GI symptoms occurred
Ye et al., (2015)	Adults;<br>36 ± 16	Resistant Maltodextrin	5 or 10 g/d	No reported GI discomfort
van den Heuvel (2004)	Adults;<br>31.7 ± 9.1	Resistant Maltodextrin	10, 30, and 60 g/day OR<br>10, 15, 45, and 80 g/d	Well tolerated up to a dose of 45 g daily. Higher daily dosages (60 and 80 g) may result in flatulence, but did not result in diarrhea.
Fastinger et al., (2008)	Adults;<br>Group I: 26.6 ± 4.5<br>Group II: 28.2 ± 6.1<br>Group III: 26.7 ± 4.2	Resistant Maltodextrin	15 g/d	Very minor effects in gastrointestinal tolerance
Vuksan et al., (2009)	Children;<br>16.1 ± 0.6	PolyGlycopleX (PGX)	5g	No differences in gastrointestinal tolerance between groups
Carabin et al., (2009)	Adults;<br>31.6 ± 10.5	PolyGlycopleX (PGX)	2.5-10 g/d	Well tolerated with only mild to moderate adverse gastrointestinal effects that did not differ from those seen in the control groups.
Ho et al., (2016)	Children aged 8 to 17 y	Maltodextrin<br>Oligofructose-enriched inulin	Placebo group:<br>(maltodextrin 3.3 g/day<br><br>Prebiotic group:<br>oligofructose-enriched inulin 8 g/d	Authors anticipate a change in gut microbiota, gut permeability and inflammatory markers over the course of several weeks of prebiotic supplementation. No GI issues anticipated
Drabińska., et al (2018)	Children	Maltodextrin	Prebiotic group:<br>oligofructose-	No reported GI discomfort

	Synergy group: 10 (range: 5–17)<br>Placebo group:<br>10 (range: 4–16)	Oligofructose-enriched inulin	enriched inulin 10 g/day<br>Placebo group: maltodextrin.	
Abrams at al., (2007)	Children aged 9 to 13 y of age	Inulin-type fructans (ITF)<br><br>Maltodextrin	Prebiotic group:<br>1:1 of oligofructose and long-chain inulin<br>8g/d<br><br>Control: maltodextrin 8 g/day	No reported GI discomfort
Cani et al., (2005)	Adults aged 21–39 y of age	Oligofructose<br><br>Maltodextrine	8 g each/ twice daily (16 g/d in total).	Compliance was excellent; minor gastrointestinal disorders (abdominal rumbling, flatulence) were reported only on the first 3 days of oligofructose treatment.
Waligora-Dupriet et al., (2017)	Oligofructose (OF) group: 14.2 ± 3.5 months of age<br><br>Maltodextrin group: 12.6 ± 3.4 months of age	Oligofructose<br><br>Maltodextrin	Oligofructose group: 2 g/d<br><br>Control: 2 g/d of maltodextrin<br><br>In one single daily administration.	No intolerance phenomenon induced any exclusion from the protocol. There were no adverse events.<br>The number of episodes of flatulence and diarrhea were significantly lower in the OF group as compared to the control group.
Moore et al., (2003)	Infants aged 4–12 months of age	Oligofructose (OF)<br><br>Maltodextrin	OF group:<br>Combination of cereal with 0.03 g OF/g<br>cereal<br><br>Control: combination of cereal with 0.03 g maltodextrin	Increase in intestinal gas and discomfort
Kranz et al., (2012)	Summary of existing evidence on implications of dietary fiber on constipation, obesity, and diabetes in children.			Fiber contributes to the maintenance of a healthy gastro-intestinal function and prevents and treats childhood constipation.
Curtin University, Australia (2019)	Aim: examine the effect of PolyGlycopleX® (PGX®) supplementation on metabolic risk factors in overweight and obese children	PGX	Seventy overweight and obese children will consume 10 g PGX® or placebo supplement (as a divided dose of 5 g) for 16 weeks.	Ongoing study
**Adaptation to improved tolerance of fiber over time**				
Grabitske et al., (2009)	Review of published studies reporting GI effects of Low-digestible carbohydrates (LDCs)	Sixty-eight studies and six review articles were evaluated		Daily intakes of 8–10 g sorbitol and xylitol and 25 g isomalt or polyglycitol may contribute to a mild laxative effect yet may be acceptable

				for school-age children and adolescents. More clinical studies of LDCs should be conducted with children to assess the effects of their incorporated into beverages and various foods consumed with meals.
Makki et al., (2018)	Review focused on dietary fibers and their impact on gut microbial ecology, host physiology, and health	Slower fermenting polysaccharides, such as resistant starch, arabinoxylan, acacia gum, and resistant maltodextrin		Daily fiber amounts > 50 g show significant improvements in health markers. Tolerance to fiber is dependent on each individual and often improves over time as the gastrointestinal tract and microbiota adapt to higher doses of dietary fiber.
Mego, Manichanh., et al (2017)	Adults, 18–54 y of age	Galactooligosaccharide prebiotic administration (HOST-G904)	2.8 g/d for 3 weeks (main study) + probe meal at interventions with 12g additional fibre	The availability of substrates induces an adaptation of the colonic microbiota activity in bacterial metabolism, which produces less gas and associated issues.
Mego, Accarino, et al., (2017)	Adults, 25-50 y of age	Galactooligosaccharide prebiotic administration (HOST-G904)	2.8 g/d for 2 weeks (ancillary study) + normal diet	Initially increased intestinal gas production and this increase declined back to baseline after 2 week administration.

Abrams SA, Griffin IJ, Hawthorne KM, Ellis KJ. Effect of prebiotic supplementation and calcium intake on body mass index. *J Pediatr*. 2007;151(3):293-298

Babiker R, Elmusharaf K, Keogh MB, Saeed AM. Effect of Gum Arabic (Acacia Senegal) supplementation on visceral adiposity index (VAI) and blood pressure in patients with type 2 diabetes mellitus as indicators of cardiovascular disease (CVD): a randomized and placebo-controlled clinical trial. *Lipids Health Dis.* 2018;17(1):56. PMID: 29558953

Babiker R, Merghani TH, Elmusharaf K, Badi RM, Lang F, Saeed AM. Effects of Gum Arabic ingestion on body mass index and body fat percentage in healthy adult females: two-arm randomized, placebo controlled, double-blind trial. *Nutr J.* 2012;11:111. PMID: 23241359

Calame W, Thomassen F, Hull S, Viebke C, Siemensma AD. Evaluation of satiety enhancement, including compensation, by blends of gum arabic. A methodological approach. *Appetite.* 2011;57(2):358-364. PMID: 21683750

Cani PD, Joly E, Horsmans Y, Delzenne NM. Oligofructose promotes satiety in healthy human: a pilot study. European Journal Of Clinical Nutrition. 2005;60:567

Carabin IG, Lyon MR, Wood S, Pelletier X, Donazzolo Y, Burdock GA. Supplementation of the diet with the functional fiber PolyGlycoplex® is well tolerated by healthy subjects in a clinical trial. *Nutr J.* 2009;8:9. PMID: 19196472

Cherbut C, Michel C, Raison V, Kravtchenko T, Severine M. Acacia Gum is a Bifidogenic Dietary Fibre with High Digestive Tolerance in Healthy Humans. *Microb Ecol Health Dis.* 2003;15(1):43-50.

Curtin University, Australia (2019). Trial ID ACTRN12617000960358. Available online at: http://www.anzctr.org.au/TrialSearch.aspx?searchTxt=PolyGlycopleX&isBasic=True

Drabińska N, Krupa-Kozak U, Abramowicz P, Jarocka-Cyrta E. Beneficial Effect of Oligofructose-Enriched Inulin on Vitamin D and E Status in Children with Celiac Disease on a Long-Term Gluten-Free Diet:

A Preliminary Randomized, Placebo-Controlled Nutritional Intervention Study. *Nutrients*. 2018;10(11):1768

Fastinger ND, Karr-Lilienthal LK, Spears JK, Swanson KS, Zinn KE, Nava GM, Ohkuma K, Kanahori S, Gordon DT, Fahey GC Jr. A novel resistant maltodextrin alters gastrointestinal tolerance factors, fecal characteristics, and fecal microbiota in healthy adult humans. *J Am Coll Nutr.* 2008;27(2):356-366. PMID: 18689571

Grabitske HA, Slavin JL. Gastrointestinal effects of low-digestible carbohydrates. *Crit Rev Food Sci Nutr*. 2009;49(4):327-360

Ho J, Reimer RA, Doulla M, Huang C. Effect of prebiotic intake on gut microbiota, intestinal permeability and glycemic control in children with type 1 diabetes: study protocol for a randomized controlled trial. Trials. 2016;17(1):347.

Holscher HD, Doligale JL, Bauer LL, Gourineni V, Pelkman CL, Fahey GC, Swanson KS. Gastrointestinal tolerance and utilization of agave inulin by healthy adults. *Food Funct*. 2014 Jun;5(6):1142-9. PMID: 24664349

Kranz S, Brauchla M, Slavin JL, Miller KB. What do we know about dietary fiber intake in children and health? The effects of fiber intake on constipation, obesity, and diabetes in children. *Adv Nutr*. 2012;3(1):47-53.

Liber A, Szajewska H. Effect of oligofructose supplementation on body weight in overweight and obese children: a randomised, double-blind, placebo-controlled trial. *Br J Nutr.* 2014;112(12):2068-2074.

Makki K, Deehan EC, **Walter J**, Backhed F. The Impact of Dietary Fiber on Gut Microbiota in Host Health and Disease. *Cell Host Microbe*. 2018;23(6):705-715

Mego M, Accarino A, Tzortzis G, Vulevic J, Gibson G, Guarner F, Azpiroz F. Colonic gas homeostasis: Mechanisms of adaptation following HOST-G904 galactooligosaccharide use in humans. Neurogastroenterology and motility : *the official journal of the European Gastrointestinal Motility Society*. 2017;29(9).

Mego M, Manichanh C, Accarino A, Campos D, Pozuelo M, Varela E, Vulevic J, Tzortzis G, Gibson G, Guarner F, Azpiroz F. Metabolic adaptation of colonic microbiota to galactooligosaccharides: a proof-of-concept-study. *Alimentary pharmacology & therapeutics*. 2017;45(5):670-680.

Nicolucci AC, Hume MP, Martinez I, Mayengbam S, **Walter J**, Reimer RA. Prebiotics Reduce Body Fat and Alter Intestinal Microbiota in Children Who Are Overweight or With Obesity. *Gastroenterology.* 2017;153(3):711-722. PMID: 28596023

Pasman W, Wils D, Saniez MH, Kardinnal A. Long-term gastrointestinal tolerance of NUTRIOSE FB in healthy men. *Eur J Clin Nutr.* 2006; 60(8):1024-34. PMID: 16482066

Pedersen C, Lefevre S, Peters V, Patterson M, Ghatei MA, Morgan LM, Frost GS. Gut hormone release and appetite regulation in healthy non-obese participants following oligofructose intake. A dose-escalation study. *Appetite.* 2013;66:44-53. PMID: 23474087

van den Heuvel EGHM, Wils D, Pasman WJ, Bakker M, Saniez MH, Kardinaal AFM. Short-term digestive tolerance of different doses of NUTRIOSE FB, a food dextrin, in adult men. *Eur J Clin Nutr.* 2004;58(7):1046-1055. PMID: 15220947

Vuksan V, Panahi S, Lyon M, Rogovik AL, Jenkins AL, Leiter AL. Viscosity of fiber preloads affects food intake in adolescents. *Nutr Metab Cardiovasc Dis*. 2009;19(7)-498-503. PMID: 19157816

Waligora-Dupriet AJ, Campeotto F, Nicolis I, Bonet A, Soulaines P, Dupont C, Butel MJ. Effect of oligofructose supplementation on gut microflora and well-being in young children attending a day care centre. *Int J Food Microbiol*. 2007;113(1):108-113.

Weber TK, Toporovski MS, Tahan S, Neufeld CB, de Morais M. Dietary fiber mixture in pediatric patients with controlled chronic constipation. *J Pediatr Gastroenterol Nutr*, 2014. 58(3):297-302. PMID: 24157445

Ye Z, Arumugam V, Haugabrooks E, Williamson P, Hendrich S. Soluble dietary fiber (Fibersol-2) decreased hunger and increased satiety hormones in humans when ingested with a meal. *Nutr Res.* 2015;35(5):393-400. PMID: 25823991
